# Indices of obesity, malnutrition and inflammation are strong predictors of mortality in adults with type 2 diabetes: the FIELD study

**DOI:** 10.1007/s00125-026-06848-6

**Published:** 2026-09-17

**Authors:** Anthony C. Keech, Rachel L. O’Connell, Andrzej S. Januszewski, Liping Li, Chris Dalton, R. John Simes, David R. Sullivan, Marja-Ritta Taskinen, Russell S. Scott, James D. Best, Alicia J. Jenkins, James D. Otvos

**Affiliations:** 1 National Health and Medical Research Council Clinical Trials Centre, Faculty of Medicine and Health, The University of Sydney, Camperdown, NSW Australia; 2 Department of Cardiology, Royal Prince Alfred Hospital, Camperdown, NSW Australia; 3 Sydney Pharmacy School, Faculty of Medicine and Health, The University of Sydney, Camperdown, NSW Australia; 4 Department of Medicine, Helsinki University Central Hospital, University of Helsinki, Helsinki, Finland; 5 Christchurch Hospital and NZ Clinical Research Ltd, Christchurch, New Zealand; 6 Lee Kong Chian School of Medicine, Nanyang Technological University, Singapore, Singapore; 7 Diabetes and Vascular Medicine, Baker Heart and Diabetes Institute, Melbourne, VIC Australia; 8 School of Medicine, University of North Carolina, Chapel Hill, NC USA

**Keywords:** Cardiovascular outcomes, Inflammation, Metabolomics, Mortality, Sarcopenia, Type 2 diabetes

## Abstract

**Aims/hypothesis:**

Metabolic risk in type 2 diabetes is not fully captured by standard clinical measures. Use of NMR-based metabolomic indices may enhance risk prediction, but their role in individuals with type 2 diabetes is unresolved.

**Methods:**

We analysed data from 9550 adults with type 2 diabetes who were followed for 5 years in the Fenofibrate Intervention and Event Lowering in Diabetes (FIELD) study. The composite index MVX (metabolic vulnerability), and its components IVX (inflammation vulnerability index) and MMX (metabolic malnutrition index), were derived from baseline plasma NMR. Associations with mortality and non-fatal events were assessed using Cox models adjusted for clinical variables and treatment allocation. Predictive performance was assessed using C statistics and variable contribution analysis.

**Results:**

All three indices were associated with total mortality. Participants in the highest MVX quintile (Q5) had an over fourfold greater unadjusted mortality risk, and more than double the risk after adjustment (HR 2.31; 95% CI 1.71, 3.12). Each standard deviation increase in MVX showed a 75% higher mortality risk (95% CI 62, 89; *p*<0.001). MVX was associated with cardiovascular and cancer mortality separately, and independently predicted non-fatal cardiovascular events and new incident cancer. MMX and IVX showed weaker and less consistent associations. MVX improved risk model discrimination (C statistic 0.725 to 0.737; *p*=0.0003) and outperformed prior CVD history as a predictor.

**Conclusions/interpretation:**

MVX independently predicts total and cardiovascular mortality in type 2 diabetes, offering prognostic value beyond traditional risk factors. The absolute event rates reflect the treatment standards of the FIELD recruitment period (1997–2000), prior to widespread use of several contemporary cardioprotective therapies. The results support further investigation of the clinical utility of MVX for determination of personalised risk.

**Graphical Abstract:**

**Figure:**
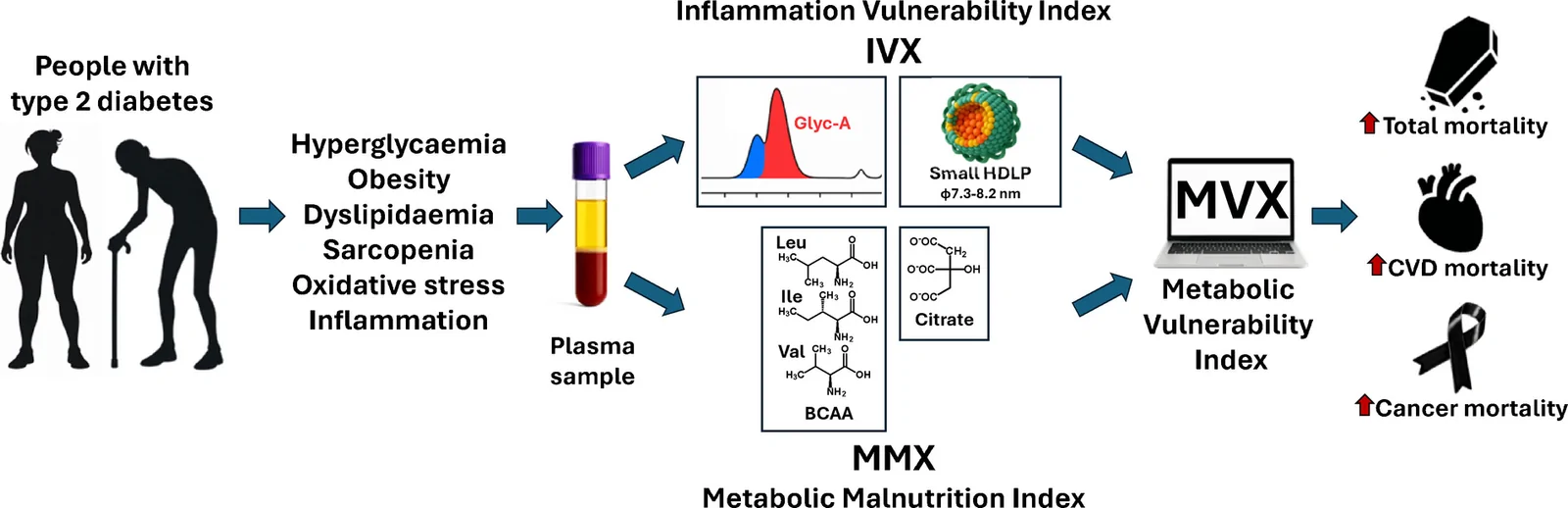

**Supplementary Information:**

The online version contains peer-reviewed but unedited supplementary material available at https://doi.org/10.1007/s00125-026-06848-6.

**Figure:**
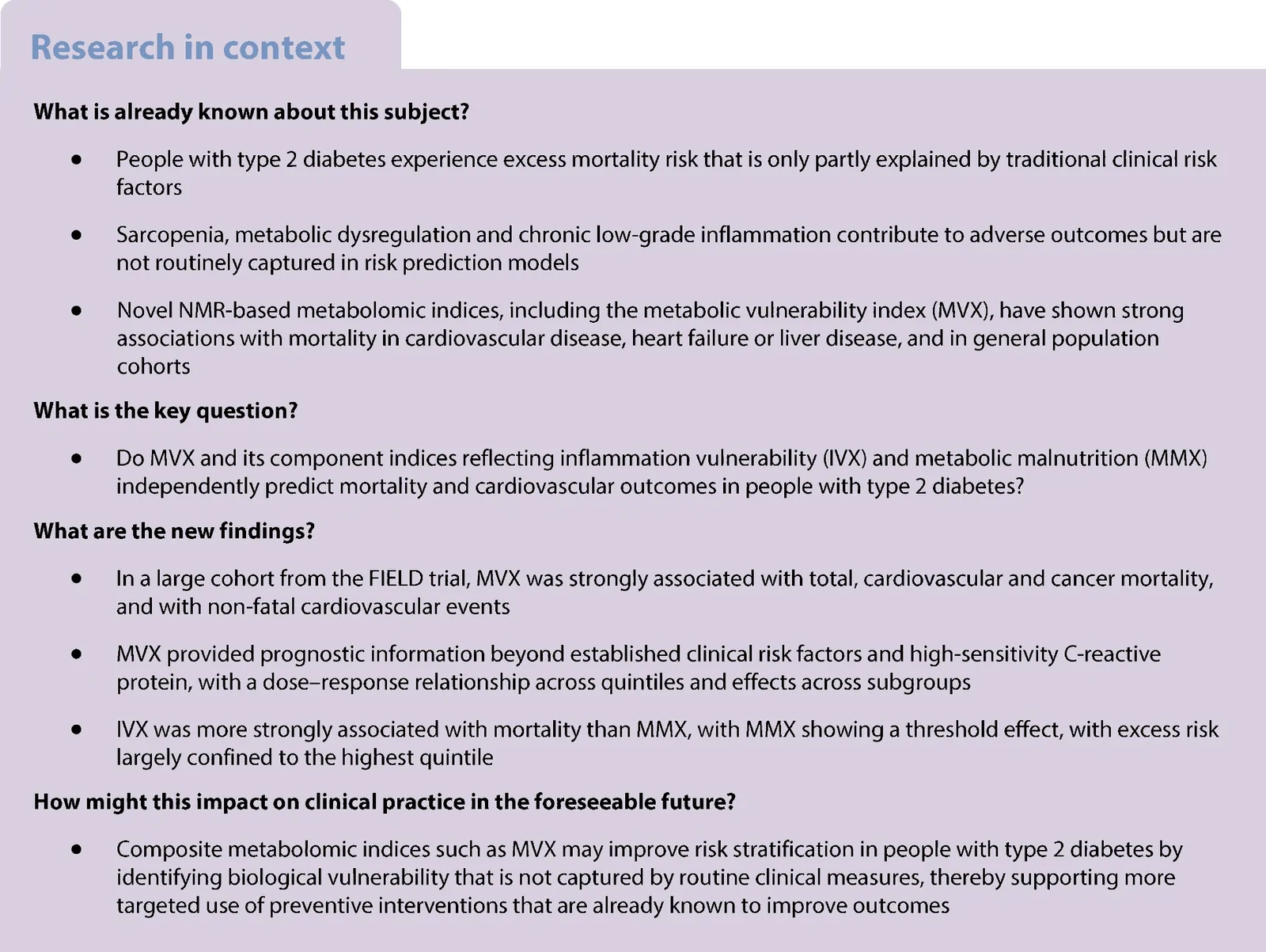

## Introduction

Dysglycaemia associated with the insulin resistance that occurs in type 2 diabetes consumes clinical time and costs. The more recent concept of malnutrition–sarcopenia or sarcopenic obesity is another important aspect of the condition that remains incompletely understood [1]. Similarly, the notion that underlying systemic inflammation is an inevitable consequence of persistent hyperglycaemia and/or obesity, and the cascade of downstream biological effects, are now well-accepted, but the drivers of inflammation beyond hyperglycaemia also await an integrated explanation [2, 3]. Chronic low-grade inflammation, mitochondrial dysfunction and altered protein turnover are increasingly recognised as inter-related processes in type 2 diabetes [4–6]. Insulin resistance promotes lipotoxicity and ectopic fat deposition, leading to oxidative stress and activation of inflammatory pathways. Simultaneously, impaired anabolic signalling and chronic inflammation accelerate muscle protein breakdown, contributing to sarcopenia and reduced metabolic reserve. Together, these processes may create a state of heightened biological vulnerability that predisposes to cardiovascular events and premature mortality.

Recently, use of a novel multimarker named MVX (metabolic vulnerability index) that reflects both metabolic malnutrition and chronic inflammation has been reported in individuals with coronary disease [7], heart failure [8] or liver disease [9] and in the general population [10–12]. MVX integrates information from six metabolomic biomarkers measured in plasma using NMR spectroscopy. MVX exhibits strong associations with mortality in these disease settings, over and above those of the classical risk factors that are used regularly for risk prediction. The efficient measurement in plasma of MVX and its component parts, IVX (inflammation vulnerability index) and MMX (metabolic malnutrition index), using high-throughput NMR provides an opportunity to evaluate the extent to which these new indices may offer fresh insights into mortality prognosis in various populations. We explored whether these measures play a similar role in people with type 2 diabetes, using data and samples from the Fenofibrate Intervention and Event Lowering in Diabetes (FIELD) trial [13]. FIELD participants were recruited in 1997–2000, and cardiovascular disease (CVD) epidemiology and management in type 2 diabetes have improved substantially since that time, potentially altering the absolute risks reported here.

## Methods

The FIELD trial was a randomised trial of the lipid-modifying drug, fenofibrate, compared with placebo among 9795 adults with type 2 diabetes who were followed for a median of 5 years [13]. The trial recruited individuals from Australia, New Zealand and Finland between 29 September 1997 and 21 July 2000. Participants were adults with type 2 diabetes recruited through participating centres in Australia, New Zealand and Finland. The cohort included both men and women and a broad range of ages, diabetes durations and clinical risk profiles, as described in Table 1. The sex of participants was self-determined, and information on gender identity was not sought. However, the trial eligibility criteria excluded some higher-risk individuals, including those with a recent myocardial infarction or stroke, and clinical trial participation may have resulted in under-representation of frailer individuals and some ethnic minority and Indigenous populations. Detailed socioeconomic information was not collected, and the regional representativeness of participants beyond the recruiting centres cannot be fully assessed. Over 70% of screened individuals were recruited into the trial. The cohort may therefore most appropriately be considered to be representative of trial-eligible adults with type 2 diabetes during the FIELD recruitment period, rather than of the entire population with type 2 diabetes. All participants provided written informed consent. The study protocol was approved by local and national ethics committees, and was undertaken in accordance with the Declaration of Helsinki and Good Clinical Practice Guidelines. Eligible participants had a type 2 diabetes diagnosis according to the 1985 WHO criteria [14], were not already taking statin therapy at screening, had a total cholesterol (TC) of 3.0–6.5 mmol/l plus either a TC/HDL-cholesterol (HDL-C) ratio of ≥4.0 or a fasting triacylglycerol level of 1.0–5.0 mmol/l, with no clear indication for statins or other lipid-modifying therapy, and had not had a myocardial infarct or stroke within the previous 3 months. All eligible participants who provided consent entered a 4-week dietary run-in phase, followed sequentially by 6 weeks of placebo and 6 weeks of fenofibrate. Participants were unaware of the treatment received during the run-in phases. Those who remained eligible and demonstrated >80% adherence during the active-treatment run-in were randomised to receive either 200 mg co-micronised fenofibrate or a matching placebo for the duration of the trial. During the randomised phase, participants, investigators and study personnel involved in clinical assessment were masked to treatment allocation, and clinical outcomes were adjudicated by an independent committee who were also masked to treatment allocation. Further details have been published previously [15]. Blood samples were drawn at screening (prior to the run-in phases) and periodically at subsequent visits. Ascertainment of endpoints was undertaken by a specific outcome assessment committee who were masked to treatment allocation, as previously described [16]. Clinical chemistry measurements, including fasting lipids, HbA_1c_, creatinine, homocysteine and high-sensitivity C-reactive protein (hsCRP), were performed using standard enzymatic and immunoassay methods in an accredited central laboratory. eGFR was calculated using the CKD-EPI 2021 creatinine equation [17].

**Table 1:** Baseline clinical characteristics of the 9550 FIELD trial participants studied

Variable	Died (*N*=672)	Alive (*N*=8878)	*p* value
Male	490 (73)	5504 (62)	<0.001
European descent	627 (93)	8228 (93)	0.548
Age at visit 1 (years)	65.8±6.2	62.0±6.8	<0.001
Diabetes duration (years)^a^	5.4 (2.8)	4.2 (2.8)	<0.001
BMI (kg/m^2^)^a^	30.0 (1.2)	30.2 (1.2)	0.408
Waist–hip ratio^a^	0.95 (1.09)	0.93 (1.09)	<0.001
Hypertension	429 (64)	4959 (56)	<0.001
Current smoker	109 (16)	794 (9)	<0.001
Chronic complications			
Previous CVD	271 (40)	1799 (20)	<0.001
Previous stroke	39 (6)	297 (3)	<0.001
Previous angina	159 (24)	992 (11)	<0.001
Peripheral vascular disease	115 (17)	578 (7)	<0.001
Coronary revascularisation^b^	54 (8)	299 (3)	<0.001
Microvascular disease	348 (52)	2835 (32)	<0.001
Retinopathy	97 (14)	697 (8)	<0.001
Neuropathy	80 (12)	461 (5)	<0.001
Nephropathy	277 (41)	2160 (24)	<0.001
Microvascular-amputation	1 (0)	17 (0)	0.806
Lipids			
TC (mmol/l)	4.95±0.79	5.04±0.69	<0.001
LDL-cholesterol (mmol/l)	3.03±0.69	3.07±0.65	0.112
HDL-C (mmol/l)	1.04±0.25	1.10±0.26	<0.001
Non-HDL-C (mmol/l)	3.91±0.75	3.94±0.68	0.278
Triacylglycerol level (mmol/l)^a^	1.80 (1.49)	1.78 (1.49)	0.428
HbA_1c_ (mmol/mol)^a^	54.6 (1.3)	51.5 (1.3)	<0.001
HbA_1c_ (%)^a^	7.2 (1.2)	6.9 (1.2)	<0.001
Plasma creatinine (µmol/l)	83.7±18.4	77.1±15.5	<0.001
eGFR (ml/min per 1.73 m^2^)	83.1±15.7	89.2±13.8	<0.001
Homocysteine (µmol/l)^a^	10.9 (1.4)	9.6 (1.3)	<0.001
hsCRP (mg/l)^a^	3.34 (3.17)	2.72 (2.95)	<0.001
NMR metrics			
GlycA (µmol/l)	467±83	445±71	<0.001
Small HDLPs (µmol/l)	15.5±2.9	16.7±2.6	<0.001
Leucine (µmol/l)	126±34	126±33	0.791
Valine (µmol/l)	241±43	242±42	0.333
Isoleucine (µmol/l)	81±20	80±19	0.218
Citrate (µmol/l)	120.8±29.7	116.1±28.1	<0.001
MVX score	52.0±8.7	47.1±8.3	<0.001
IVX score	48.6±11.5	42.5±10.8	<0.001
MMX score	55.3±6.2	54.0±5.8	<0.001

Values for categorical variables are *n* (%); those for continuous variables are mean ± SD, unless specified otherwise

^a^Geometric means (geometric SD factor); values were log_e_-transformed prior to analysis

^b^By percutaneous transluminal coronary angioplasty or coronary artery bypass grafting

*p* values were calculated using the χ^2^ test for categorical variables or Student’s *t* test for continuous variables

### NMR analyses

EDTA-anticoagulated plasma from blood drawn at the screening visit in the fasting state was stored at −80°C until analysis in 2016 using NMR LipoProfile technology on the NMR Profiler platform at LipoScience (now Labcorp, Morrisville, NC, USA), employing the LP4 algorithm [18, 19]. The LipoScience (Labcorp) NMR platform was selected because the MVX, IVX and MMX indices had been derived and validated using this platform and the proprietary LP4 algorithm. Use of the same analytical platform ensured methodological consistency and comparability with prior studies. All analyses were performed in a central laboratory regulated by the US Clinical Laboratory Improvement Amendments whose staff were blinded to clinical outcomes. Analyses were performed using standardised clinical operating procedures that included daily calibration and monitoring of quality control specimens in each run to minimise batch effects. MVX scores (1–100) were calculated by Labcorp using NMR-measured concentrations of the following six metabolomic biomarkers quantified as previously described: GlycA [20], small HDL particles (HDLPs) <8.8 nm diameter [7, 21], citrate [22], and three branched-chain amino acids (BCAAs): valine, leucine and isoleucine [23]. The two component parts of MVX that are speculated to represent the inflammatory and metabolic malnutrition contributions were also calculated: IVX, comprising GlycA plus small HDLPs, and MMX, comprising citrate, valine, leucine and isoleucine [7]. To correct for sex differences in metabolite levels unrelated to mortality risk that would make the index scores several points higher in women, the original equations [7] for calculation of MVX, IVX and MMX used sex-specific weighting factors to equalise distributions in men and women. As the index values used in this study were calculated by Labcorp using equations that were not sex-specific [10], we corrected the resulting sex imbalances by reducing the MVX, IVX and MMX scores of female participants by 5.2, 5.6 and 2.1 points, respectively, to equalise median values in men and women. Index scores and the metabolite levels used for calculation have been validated by Labcorp as stable in EDTA-anticoagulated plasma when frozen at <−70°C for at least 12 years [8]. The inter-assay precision (coefficient of variation) for the three indices is <5%.

### Statistical analysis

Values for continuous variables are presented as means ± SD or median (IQR), depending on their distribution, and those for categorical variables are presented as *n* (%). The χ^2^ statistic and unpaired Student’s *t* test were used to compare baseline characteristics among those who did or did not die during follow-up (a median of 5 years). Cumulative mortality incidence curves for subgroups of baseline IVX, MMX and MVX values were reported. All results are presented according to quintiles of the overall population for each multimarker. No formal adjustment for multiple comparisons was performed because the analyses were hypothesis-driven and focused on three pre-specified metabolomic indices.

We assessed the associations of MVX, IVX and MMX multimarker scores with total mortality, death due to CVD and death due to cancer using Cox proportional hazards models adjusted for baseline age, diabetes duration, sex, ethnicity, hypertension, smoking status, prior history of CVD, eGFR, hsCRP, BMI, TC, HDL-C, triacylglycerol level, study treatment allocation and baseline HbA_1c_ levels.

The proportional hazards assumption was assessed using the Harrell workflow [24], by examining scaled Schoenfeld residuals for each predictor and inspecting smoothed residual plots. No meaningful departures from proportional hazards were observed. Interactions were not tested as the combined multimarkers were calculated using interactions of the individual metabolites.

Estimates of the relative importance of each predictive variable were based on its χ^2^ value calculated using the Harrell *rms* (regression modelling strategies) package in R. The models were validated by calculating Harrell’s C index for the MVX model on overall mortality [24]. Differences between model discrimination were assessed by comparing paired Harrell C statistics using the Gonen–Heller test implemented in the R *compareC* package. In sensitivity analysis, optimism-adjusted C statistics (with 1000 bootstraps) were also calculated [25]. All reported *p* values are two-sided, and significance was taken at *p*<0.05.

### Missing data

The proportion of missing data was extremely low for most covariates. hsCRP and BMI had missing values in 1.2% and 0.06% of participants, respectively. Patterns of missingness were not associated with outcome status or levels of the three indices, and therefore data were concluded to be missing at random. Given the low proportion of missingness, analyses were conducted using complete-case models without imputation [26]. Sensitivity analyses excluding hsCRP yielded similar effect estimates (data not shown).

## Results

### Participants

Samples were available for analysis from 9550 (97.5%) of the FIELD trial participants (Table 1). Over a median of 5.0 years of follow-up, there were 672 deaths from any cause, 1267 cardiovascular events (of which 262 were fatal), 749 new cancers (of which 314 were fatal), and 96 deaths due to causes other than CVD or cancer, including 35 respiratory deaths and 23 other deaths (including trauma).

Participants who died during follow-up were older, had longer diabetes duration, higher waist–hip ratio, and a greater prevalence of clinical risk factors including hypertension, prior CVD, stroke, angina and nephropathy, compared with survivors (Table 1, all *p*<0.001). They also had significantly higher HbA_1c_ levels, plasma creatinine, homocysteine and hsCRP, and lower HDL-C and eGFR (all *p*<0.001). Importantly, all NMR-derived metabolomic indices (MVX, IVX, MMX) and the concentration of the contributing metabolites GlycA and citrate were significantly elevated among those who died, while the concentration of small HDLPs was lower. The concentrations of the BCAAs valine, leucine and isoleucine were not different between those who died and those who did not.

### NMR multimarker indices

**Fig. 1:**
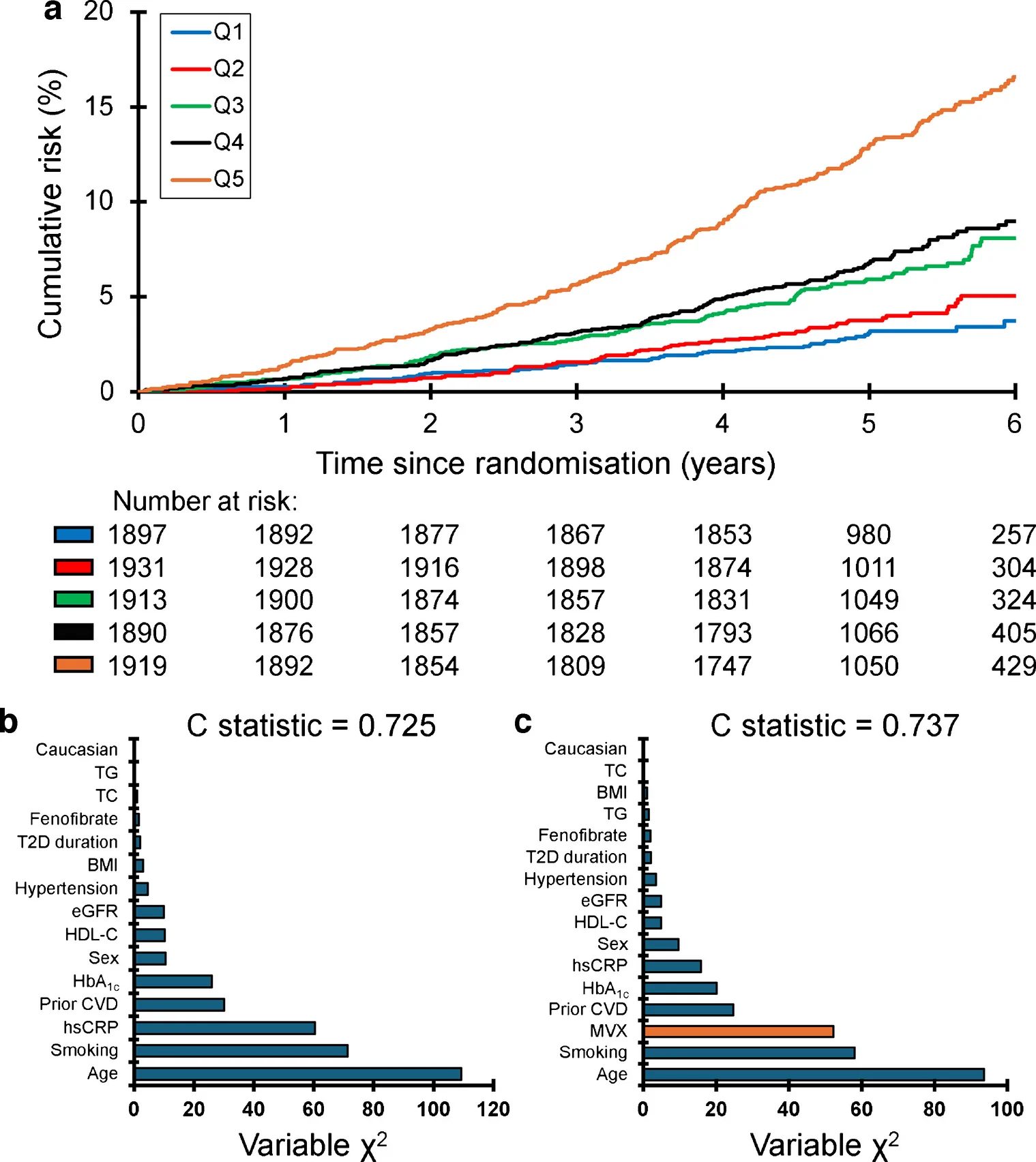
(**a**) Cumulative incidence of total mortality stratified by MVX quintiles. (**b**) Contribution of baseline clinical variables to the base model predicting total mortality, quantified by individual χ^2^ statistics. (**c**) Change in variable contributions after inclusion of MVX in the model, illustrating the added prognostic value of MVX alongside clinical covariates. The graphs in (**b**) and (**c**) show the relative contribution of individual variables to the total χ^2^ statistic from Cox proportional hazards models predicting total mortality. Variables are ranked by their χ^2^ value, illustrating the incremental explanatory value of MVX relative to established clinical predictors. The number of participants included in multivariate models was 9425, after exclusion of 119 participants with missing hsCRP values and six participants with missing BMI values. T2D, type 2 diabetes; TG, triacylglycerol

**Fig. 2:**
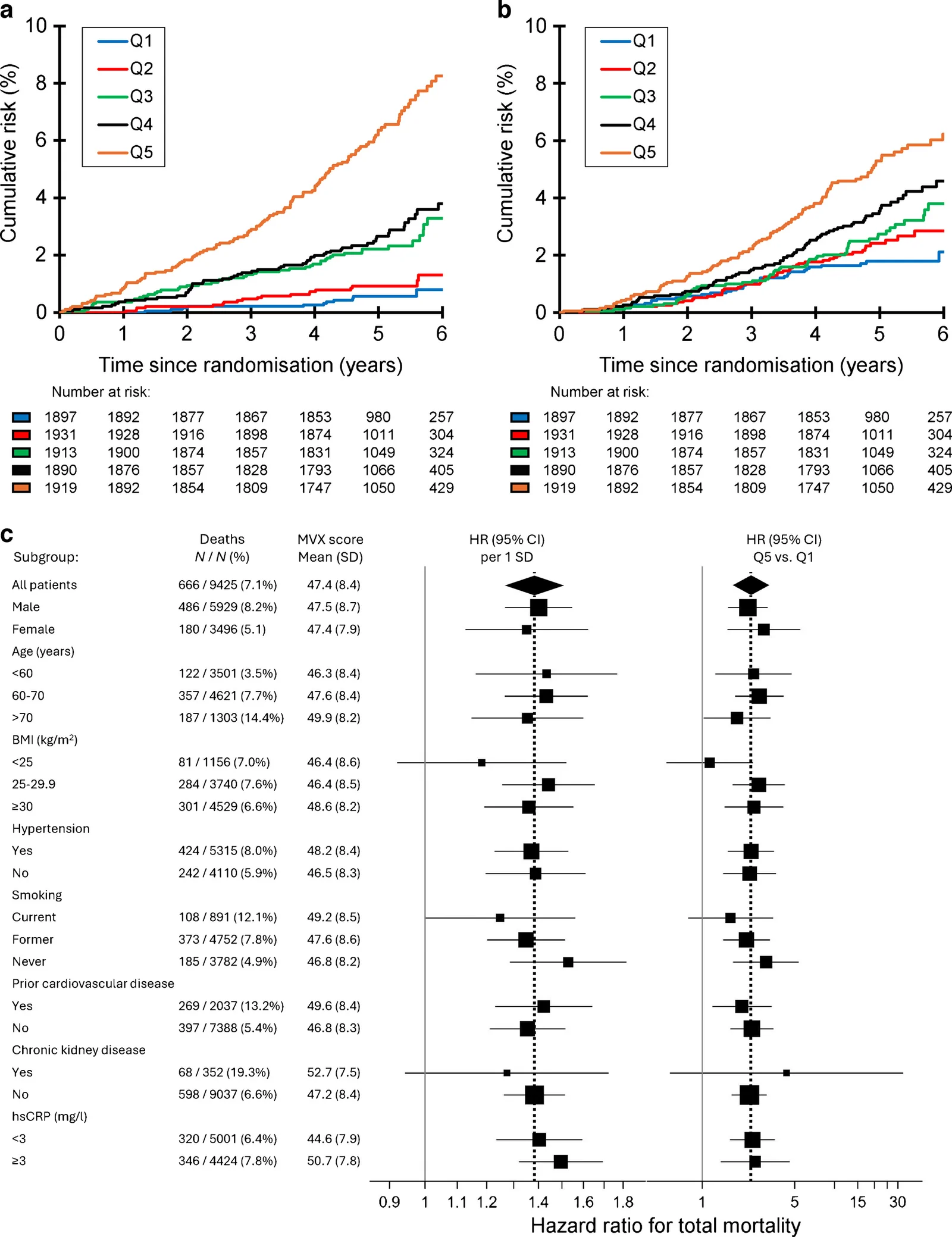
Cumulative incidence of cause-specific outcomes stratified by MVX quintiles. (**a**, **b**) Mortality due to CVD (**a**) and cancer (**b**). (**c**) Hazard ratios for total mortality per 1 SD increase in MVX and for MVX quintile 5 vs quintile 1 across predefined clinical subgroups. Hazard ratios and their 95% CIs are shown per 1 SD increase in MVX and for quintile 5 vs quintile 1, derived from Cox proportional hazards models. Models were adjusted for age, sex, race, smoking, hypertension, prior CVD, BMI, TC, HDL-C, triacylglycerol, HbA_1c_, eGFR, diabetes duration, hsCRP and fenofibrate treatment. In subgroup analyses, the stratifying variable was omitted from the adjustment set. CKD was defined as an eGFR <60 ml/min per 1.73 m^2^. The dotted vertical black line indicates the HR for all participants, and is shown as a reference to facilitate comparison of subgroup estimates with the overall effect. The number of participants included in multivariate models was 9425, after exclusion of 119 participants with missing hsCRP values and six participants with missing BMI values

MVX, IVX and MMX indices were all significantly associated with total mortality, CVD mortality and cancer death (Table 2). Of the three indices, MVX demonstrated the strongest association with mortality (Table 2, Figs 1a and 2a, b). The HRs for mortality increased progressively, being significantly elevated in the third, fourth and fifth MVX quintiles compared with the reference (Q1 [first quintile]), in both unadjusted models and those adjusted for a broad range of clinical characteristics, including glycaemia, hsCRP and treatment allocation. Further adjustment for known retinopathy at baseline did not materially alter these associations (data not shown). The clearest risk gradient was observed when comparing quintiles 1 and 2 with quintiles 3, 4 and 5 (Fig. 1a). There was a particularly high risk in the top quintile, being more than fourfold higher than in Q1 and still twice as great as Q1 after extensive adjustment. For each SD increase in the MVX score, the mortality risk was 75% higher (95% CI 62, 89; *p*<0.001). Compared with the risk model that included clinical variables (Fig. 1b; C statistic 0.725), addition of MVX improved risk prediction substantially (Fig. 1c; C statistic 0.737; *p*=0.0003). In sensitivity analyses, the optimism-adjusted C statistics were 0.719 and 0.731 respectively (only marginally smaller), with an identical difference of 0.012, indicating that the findings are robust. The pattern remained the same among 5233 individuals considered to be at lower risk (with no prior macrovascular or microvascular disease at study entry) (electronic supplementary material [ESM] Table 1).

**Table 2:** Associations of MVX, IVX and MMX with fatal and non-fatal events in the FIELD study

	First quintile	Second quintile	Third quintile	Fourth quintile	Fifth quintile	HR per SD^a^
MVX score	≤40.3	40.4–45.3	45.4–49.5	49.6–54.5	≥54.6	
Total mortality						
Events	60/1897 (3.2)	77/1931 (4.0)	125/1913 (6.5)	141/1890 (7.5)	269/1919 (14.0)	
Unadjusted HR	1.00 (reference)	1.25 (0.89, 1.75)	2.05 (1.50, 2.79)	2.31 (1.71, 3.13)	4.44 (3.36, 5.88)	1.75 (1.62, 1.89)
Adjusted HR	1.00 (reference)	1.04 (0.74, 1.46)	1.53 (1.12, 2.09)	1.51 (1.11, 2.06)	2.31 (1.71, 3.12)	1.38 (1.26, 1.51)
CVD death						
Events	12/1897 (0.6)	19/1931 (1.0)	47/1913 (2.5)	57/1890 (3.0)	127/1919 (6.6)	
Unadjusted HR	1.00 (reference)	1.54 (0.75, 3.17)	3.84 (2.04, 7.24)	4.66 (2.50, 8.69)	10.44 (5.78, 18.88)	2.14 (1.89, 2.42)
Adjusted HR	1.00 (reference)	1.22 (0.59, 2.52)	2.68 (1.41, 5.08)	2.63 (1.39, 4.95)	4.46 (2.41, 8.26)	1.59 (1.38, 1.83)
Cancer death						
Events	36/1897 (1.9)	47/1931 (2.4)	58/1913 (3.0)	71/1890 (3.8)	102/1919 (5.3)	
Unadjusted HR	1.00 (reference)	1.27 (0.83, 1.97)	1.59 (1.05, 2.41)	1.96 (1.31, 2.92)	2.83 (1.93, 4.14)	1.46 (1.30, 1.63)
Adjusted HR	1.00 (reference)	1.08 (0.70, 1.68)	1.21 (0.79, 1.85)	1.36 (0.90, 2.06)	1.66 (1.09, 2.51)	1.21 (1.07, 1.38)
Non-fatal CVD						
Events	189/1897 (10.0)	228/1931 (11.8)	213/1913 (11.1)	294/1890 (15.6)	330/1919 (17.2)	
Unadjusted HR	1.00 (reference)	1.19 (0.98, 1.44)	1.12 (0.92, 1.36)	1.59 (1.33, 1.91)	1.80 (1.51, 2.16)	1.25 (1.18, 1.33)
Adjusted HR	1.00 (reference)	1.10 (0.90, 1.34)	0.96 (0.79, 1.18)	1.27 (1.04, 1.54)	1.19 (0.97, 1.46)	1.08 (1.01, 1.15)
Non-fatal cancer						
Events	82/1897 (4.3)	92/1931 (4.8)	85/1913 (4.4)	103/1890 (5.4)	107/1919 (5.6)	
Unadjusted HR	1.00 (reference)	1.10 (0.82, 1.48)	1.03 (0.76, 1.39)	1.25 (0.93, 1.67)	1.31 (0.98, 1.75)	1.11 (1.01, 1.22)
Adjusted HR	1.00 (reference)	1.01 (0.75, 1.37)	0.92 (0.67, 1.25)	1.06 (0.78, 1.44)	1.02 (0.74, 1.41)	1.02 (0.92, 1.13)
IVX score	≤33.6	33.7–40	40.1–45.5	45.6–52.1	≥52.2	
Total mortality						
Events	62/1923 (3.2)	89/1901 (4.7)	113/1913 (5.9)	164/1908 (8.6)	244/1905 (12.8)	
Unadjusted HR	1.00 (reference)	1.45 (1.05, 2.00)	1.82 (1.33, 2.48)	2.65 (1.98, 3.55)	4.00 (3.02, 5.28)	1.69 (1.56, 1.82)
Adjusted HR	1.00 (reference)	1.21 (0.88, 1.68)	1.37 (1.00, 1.87)	1.77 (1.31, 2.39)	2.15 (1.59, 2.91)	1.36 (1.25, 1.49)
CVD death						
Events	14/1923 (0.7)	27/1901 (1.4)	44/1913 (2.3)	65/1908 (3.4)	112/1905 (5.9)	
Unadjusted HR	1.00 (reference)	1.94 (1.02, 3.70)	3.13 (1.71, 5.71)	4.63 (2.60, 8.24)	8.08 (4.64, 14.08)	1.99 (1.76, 2.25)
Adjusted HR	1.00 (reference)	1.55 (0.81, 2.95)	2.16 (1.18, 3.96)	2.61 (1.45, 4.72)	3.50 (1.95, 6.28)	1.50 (1.30, 1.73)
Cancer death						
Events	37/1923 (1.9)	48/1901 (2.5)	57/1913 (3.0)	79/1908 (4.1)	93/1905 (4.9)	
Unadjusted HR	1.00 (reference)	1.31 (0.85, 2.01)	1.54 (1.02, 2.33)	2.15 (1.45, 3.18)	2.57 (1.75, 3.76)	1.45 (1.30, 1.63)
Adjusted HR	1.00 (reference)	1.09 (0.71, 1.69)	1.17 (0.77, 1.78)	1.49 (0.99, 2.24)	1.53 (1.00, 2.33)	1.24 (1.08, 1.42)
Non-fatal CVD						
Events	184/1923 (9.6)	222/1901 (11.7)	234/1913 (12.2)	290/1908 (15.2)	324/1905 (17.0)	
Unadjusted HR	1.00 (reference)	1.23 (1.01, 1.50)	1.30 (1.07, 1.57)	1.64 (1.36, 1.97)	1.85 (1.55, 2.22)	1.26 (1.19, 1.33)
Adjusted HR	1.00 (reference)	1.13 (0.93, 1.38)	1.12 (0.92, 1.37)	1.31 (1.08, 1.60)	1.31 (1.06, 1.61)	1.12 (1.04, 1.19)
Non-fatal cancer						
Events	79/1923 (4.1)	103/1901 (5.4)	86/1913 (4.5)	89/1908 (4.7)	112/1905 (5.9)	
Unadjusted HR	1.00 (reference)	1.33 (0.99, 1.78)	1.10 (0.81, 1.49)	1.14 (0.84, 1.54)	1.46 (1.09, 1.94)	1.10 (1.01, 1.21)
Adjusted HR	1.00 (reference)	1.24 (0.92, 1.67)	0.98 (0.72, 1.35)	1.00 (0.73, 1.38)	1.17 (0.85, 1.63)	1.02 (0.91, 1.13)
MMX score	≤49.2	49.3–52.4	52.5–55.3	55.4–58.8	≥58.9	
Total mortality						
Events	102/1916 (5.3)	131/1922 (6.8)	118/1903 (6.2)	124/1887 (6.6)	197/1922 (10.2)	
Unadjusted HR	1.00 (reference)	1.28 (0.99, 1.66)	1.15 (0.88, 1.50)	1.22 (0.94, 1.58)	1.90 (1.50, 2.42)	1.21 (1.12, 1.30)
Adjusted HR	1.00 (reference)	1.18 (0.91, 1.53)	1.08 (0.82, 1.41)	1.02 (0.78, 1.33)	1.51 (1.18, 1.93)	1.13 (1.04, 1.22)
CVD death						
Events	32/1916 (1.7)	43/1922 (2.2)	47/1903 (2.5)	57/1887 (3.0)	83/1922 (4.3)	
Unadjusted HR	1.00 (reference)	1.34 (0.85, 2.12)	1.46 (0.93, 2.29)	1.78 (1.16, 2.75)	2.55 (1.70, 3.84)	1.35 (1.20, 1.51)
Adjusted HR	1.00 (reference)	1.16 (0.73, 1.85)	1.30 (0.82, 2.05)	1.35 (0.86, 2.11)	1.90 (1.25, 2.90)	1.25 (1.11, 1.42)
Cancer death						
Events	55/1916 (2.9)	69/1922 (3.6)	55/1903 (2.9)	52/1887 (2.8)	83/1922 (4.3)	
Unadjusted HR	1.00 (reference)	1.25 (0.88, 1.79)	1.00 (0.69, 1.45)	0.95 (0.65, 1.39)	1.49 (1.06, 2.10)	1.09 (0.98, 1.22)
Adjusted HR	1.00 (reference)	1.18 (0.83, 1.69)	0.96 (0.66, 1.40)	0.84 (0.57, 1.24)	1.26 (0.88, 1.78)	1.03 (0.92, 1.16)
Non-fatal CVD						
Events	236/1916 (12.3)	259/1922 (13.5)	246/1903 (12.9)	250/1887 (13.2)	263/1922 (13.7)	
Unadjusted HR	1.00 (reference)	1.10 (0.93, 1.32)	1.04 (0.87, 1.25)	1.07 (0.90, 1.28)	1.12 (0.94, 1.33)	1.04 (0.99, 1.10)
Adjusted HR	1.00 (reference)	1.02 (0.86, 1.22)	0.93 (0.78, 1.12)	0.91 (0.76, 1.10)	0.92 (0.77, 1.10)	0.98 (0.92, 1.04)
Non-fatal cancer						
Events	89/1916 (4.6)	94/1922 (4.9)	85/1903 (4.5)	113/1887 (6.0)	88/1922 (4.6)	
Unadjusted HR	1.00 (reference)	1.05 (0.79, 1.41)	0.95 (0.70, 1.27)	1.28 (0.97, 1.69)	0.97 (0.73, 1.31)	1.04 (0.95, 1.13)
Adjusted HR	1.00 (reference)	1.01 (0.75, 1.35)	0.91 (0.67, 1.23)	1.19 (0.90, 1.58)	0.90 (0.66, 1.21)	1.01 (0.92, 1.11)

Values are *n*/*N* (%) for events, and HR (95% CI) for adjusted and unadjusted HRs

^a^The SD of each score is 11.0 for IVX, 5.9 for MMX and 8.4 for MVX

HRs and 95% CIs are from Cox proportional hazard regression models. The adjusted model includes the following covariates: fenofibrate treatment, age, sex, race (European descent vs other), smoking (current, former, never), hypertension, BMI, TC, HDL-C, triacylglycerol level, HbA_1c_, eGFR, prior CVD, hsCRP and diabetes duration

Notably, in the base model without MVX (Fig. 1b), the third greatest risk contributor (after age and smoking) was the clinical inflammatory marker hsCRP. When IVX, the putative inflammatory component of MVX, was added to the base model, it became the third greatest contributor, attenuating the importance of hsCRP (ESM Fig. 1a). Similarly, in a model that included the six NMR metabolites used to calculate MVX, the predictive importance of hsCRP was exceeded by both GlycA and small HDLPs (the inflammatory biomarker constituents of IVX) (ESM Fig. 1b).

An increase of one SD in MVX was consistently associated with a significantly elevated hazard of death in all subgroups analysed, defined by sex, age, BMI, hypertension, smoking status, history of CVD, history of chronic kidney disease (CKD: defined as eGFR <60 ml/min per 1.73 m^2^) and hsCRP level (Fig. 2c). In the 352 (3.7%) participants with CKD (eGFR <60 ml/min per 1.73 m^2^), MVX was significantly associated with mortality in univariate analysis (HR 1.46 per SD; *p*=0.006), but this association was attenuated and did not remain statistically significant after multivariable adjustment (HR 1.27 per SD; *p*=0.12) in this very small sample. There was also a lack of statistical significance in the relatively small subset of individuals with low BMI (<25 kg/m^2^).

**Fig. 3:**
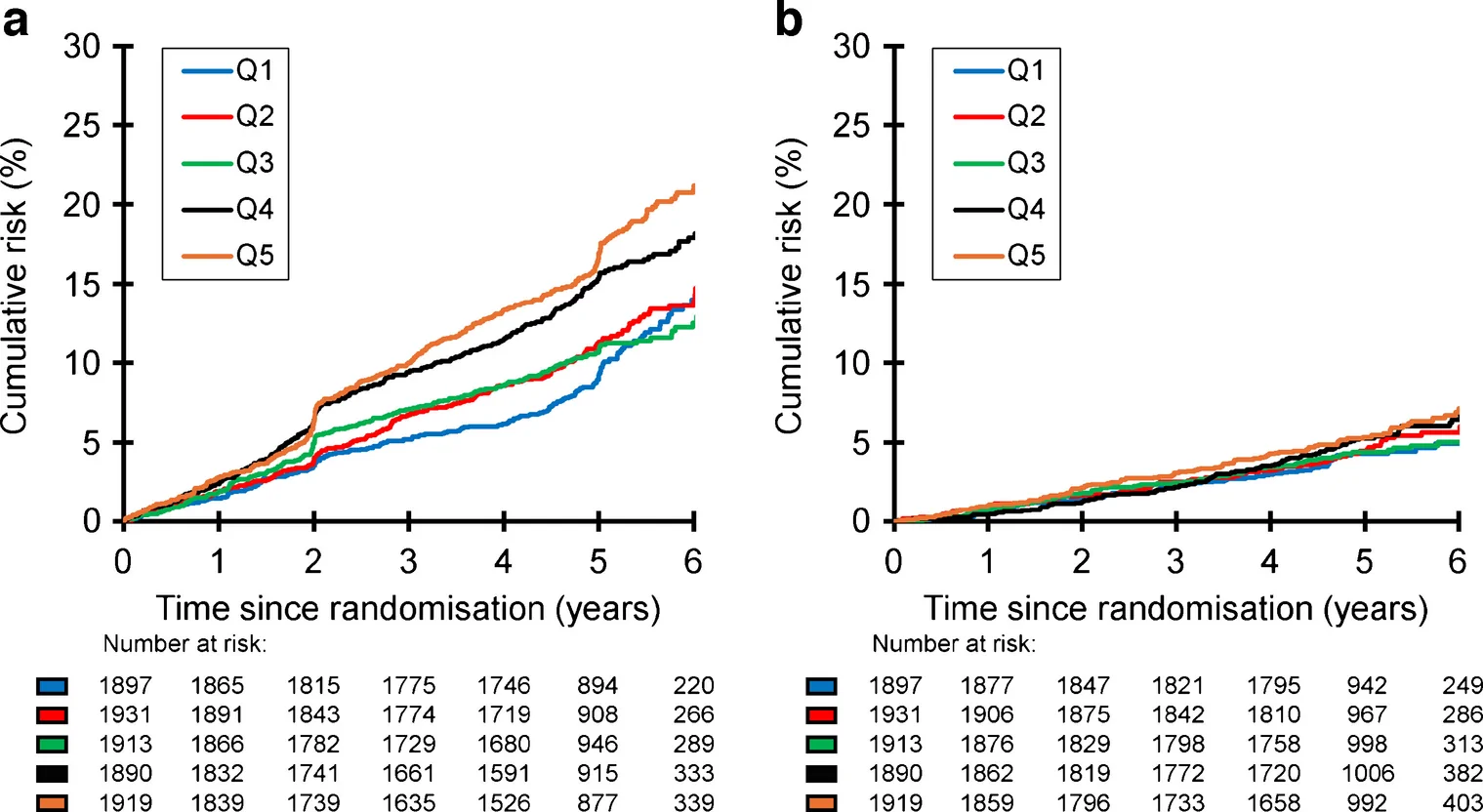
Cumulative incidence of cause-specific outcomes stratified by MVX quintiles: (**a**) non-fatal cardiovascular events, and (**b**) non-fatal cancer events

MVX was also significantly associated with the risk of non-fatal cardiovascular events, including silent myocardial infarction, which represented over 36% of cardiac events [27] in the FIELD trial (Table 2, Fig. 3a). The risk increased by 25% per SD of MVX in the unadjusted model (HR 1.25; 95% CI 1.18, 1.33) and remained significant after full adjustment for clinical covariates (HR 1.08; 95% CI 1.01, 1.15). MVX showed a much weaker relationship with incident non-fatal cancer events (Table 2, Fig. 3b), with a modest association observed in the unadjusted model (HR 1.11; 95% CI 1.01, 1.22), which was attenuated and no longer significant after full adjustment (HR 1.02; 95% CI 0.92, 1.13).

Similar patterns were also observed for IVX, with progressive increases in risk of death across quintiles of IVX with significant associations across all quintiles in the unadjusted model; however, in the adjusted model, only the fourth and fifth quintiles remained independently significant (Table 2). For each SD increase in the IVX score the risk of death was 69% higher (95% CI 56, 82; *p*<0.001).

In contrast, the risk gradients for MMX were less steep, with only participants in the highest quintile showing an independently significant higher risk of mortality in both unadjusted and adjusted models (Table 2), suggesting a possible threshold effect among people with established diabetes. For each SD increase in the MMX score, the risk was 21% higher (95% CI 12, 30; *p*<0.001). Of interest, MMX was significantly associated with mortality among the 1320 individuals over 70 years of age (ESM Table 2), and the risk contributions of MMX and its valine and isoleucine components were substantially greater in these individuals than in the younger participant subgroup (ESM Fig. 2). MMX showed no significant association with non-fatal cardiovascular events or incident cancer, in either unadjusted or adjusted models (Table 2). For all three indices, MVX, MMX and IVX, associations with increased total mortality remained present on a continuous basis (per SD) in both unadjusted and adjusted models, confirming a dose–response relationship.

**Fig. 4:**
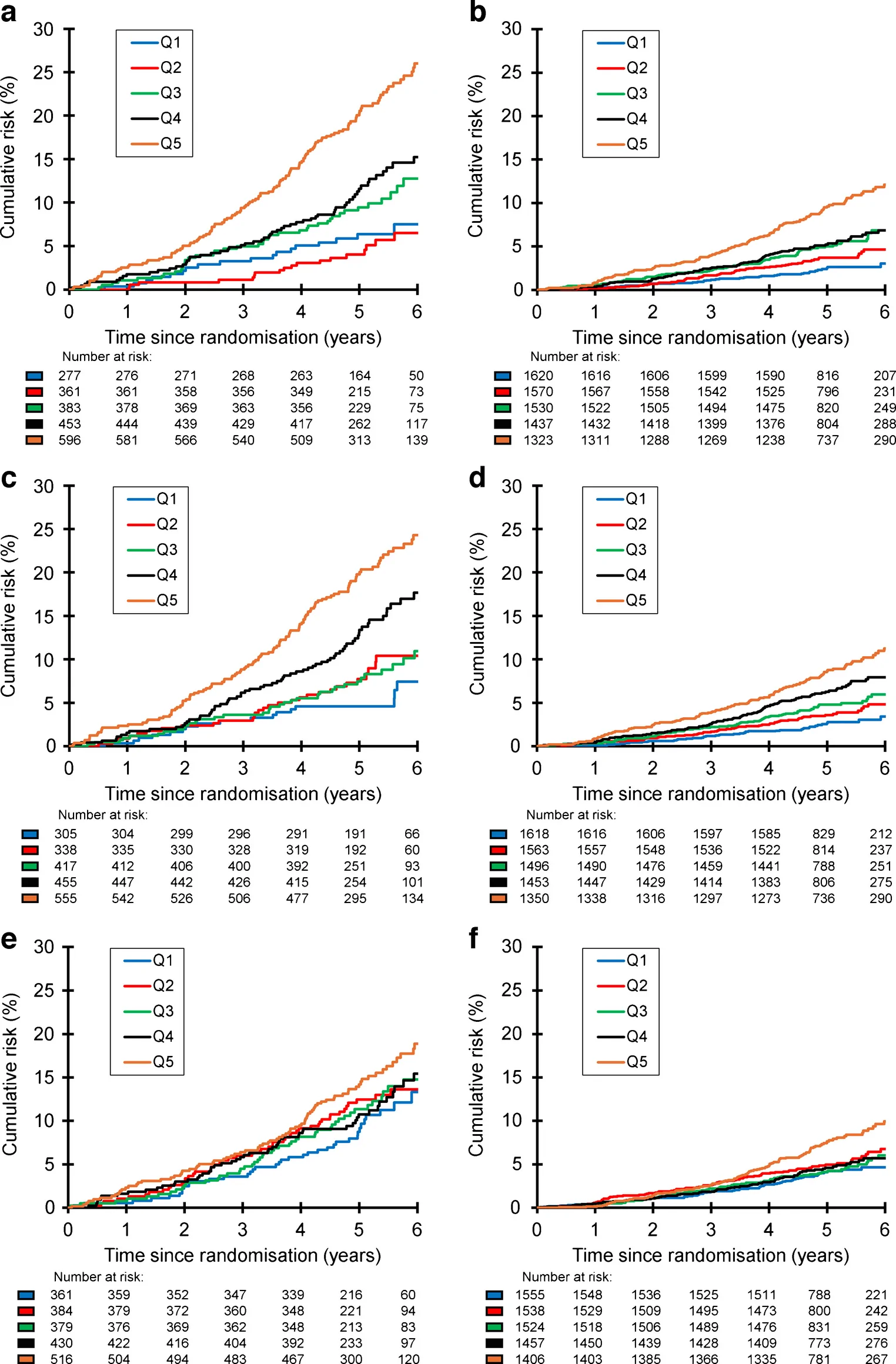
Cumulative incidence of total mortality stratified by MVX, IVX and MMX quintiles in the entire cohort, presented separately for participants with and without prior CVD, over a median follow-up duration of 5 years in the FIELD study. (**a**, **b**) MVX quintiles in participants with (**a**) and without (**b**) a history of CVD at baseline. (**c**, **d**) IVX quintiles in participants with (**c**) and without (**d**) a history of CVD at baseline. (**e**, **f**) MMX quintiles in participants with (**e**) and without (**f**) a history of CVD at baseline. Participants in higher quintiles experienced progressively greater mortality risk, regardless of prior CVD status, with absolute risks consistently lower among those without baseline CVD. The models support the existence of a dose–response relationship across quintiles that is particularly evident between the lowest (Q1) and highest (Q5) categories

The associations between total mortality and increasing quintiles of MVX, IVX and MMX were evident in both participants with and without a history of CVD at baseline (Fig. 4). While absolute event rates were higher among those with prior CVD, the risk gradients across quintiles – particularly between the lowest and highest quintiles (Q1 vs Q5) – remained consistent in both subgroups, indicating that the prognostic utility of these indices is independent of baseline CVD status.

Despite a very weak linear correlation between quintile rankings of IVX and MMX (Pearson’s *r*=0.03, *p*=0.009), a χ^2^ test demonstrated a statistically significant association between their distributions (*p*=0.008), indicating that the observed frequencies across quintile categories differed from what would be expected under complete independence. This non-random pattern is illustrated in the heatmap shown in ESM Fig. 3.

## Discussion

We conducted a post hoc analysis of the novel NMR-derived index MVX and its component indices IVX and MMX in 9550 FIELD trial participants with type 2 diabetes, the first such analysis in individuals with type 2 diabetes. Our results demonstrate strong associations of these indices of metabolic vulnerability with total mortality as well as cardiovascular and cancer mortality in adults with type 2 diabetes. These findings validate those obtained in other disease settings [7–9] and the general population [10–12], showing MVX to be a powerful independent predictor of mortality, and extend the generalisability of these observations to trial-eligible adults with type 2 diabetes, beyond traditional risk factors for fatal outcomes. The predictive contribution of MVX was strong compared with hsCRP, prior CVD and other accepted markers of poor prognosis, ranking behind only age and current smoking as a contributor to risk of overall and cause-specific death.

It should be noted that the absolute event rates observed in the FIELD trial reflect an earlier treatment era, prior to evidence for the use of routine statin therapy for individuals with type 2 diabetes and before the advent of sodium–glucose co-transporter-2 (SGLT2) inhibitors and glucagon-like peptide-1 (GLP-1) receptor agonists, and therefore may not directly translate to contemporary populations. While sarcopenia and chronic inflammation can be identified clinically or through targeted instrumental assessments, MVX provides a quantitative and integrated measure of metabolic and inflammatory perturbation at the molecular level. These subclinical metabolic and inflammatory signals may precede overt manifestations detectable by physical examination or routine laboratory testing. Accordingly, MVX should be viewed as complementary to, rather than redundant with, conventional clinical evaluation.

Our findings confirm that well-established clinical factors, i.e. older age, longer diabetes duration and history of cardiovascular or renal disease, remain among the strongest predictors of mortality in people with type 2 diabetes [28, 29]. Participants who died during follow-up were older on average, with a longer duration of diabetes, and were far more likely to have pre-existing hypertension, coronary disease, stroke, nephropathy or microvascular complications (all *p*<0.001). These observations mirror those in earlier cohort studies, in which each decade of age and each 5-year increment in diabetes duration conferred a 20–30% higher risk of death, and in which baseline CVD or reduced eGFR consistently doubled long-term mortality risk [30, 31]. Markers of poor metabolic control and systemic inflammation (higher HbA_1c_, homocysteine and hsCRP) alongside dyslipidaemia (lower HDL-C) and renal impairment (elevated plasma creatinine and reduced eGFR) further distinguished those who died from those who did not, underscoring the multifactorial pathophysiology driving adverse outcomes. These variables underpin widely used risk engines such as UKPDS [32] and ADVANCE [33], and guideline-recommended treatment targets [34], yet, even with their inclusion, our clinical-only model achieved a C statistic of 0.725, indicating that substantial residual risk is unaccounted for. Addition of MVX increased discrimination modestly but significantly (C statistic 0.725–0.737; *p*=0.0003), indicating that MVX contributes prognostic information beyond established clinical predictors rather than replacing them. In all clinical subgroups, the point estimate remained directionally consistent, suggesting that limited statistical power may have impacted the ability to detect any significance for low BMI and CKD.

FIELD participants were recruited between 1997 and 2000, prior to evidence for the widespread use of statins in individuals with type 2 diabetes and newer contemporary cardioprotective therapies, such as SGLT2 inhibitors and GLP-1 agonists. As a consequence, secular improvements in cardiovascular prevention have reduced event rates in modern type 2 diabetes populations, and risk models derived from earlier cohorts may overestimate absolute risk when applied to contemporary settings [35]. Accordingly, the absolute event rates observed in the FIELD trial should be interpreted within their historical context. Nevertheless, the biological pathways reflected by MVX (chronic inflammation and metabolic perturbation) are central to type 2 diabetes pathophysiology, suggesting that the index may capture vulnerability that persists despite advances in treatment, although external validation in other contemporary cohorts is warranted.

Use of the metabolomic indices extends the current understanding of mortality risk in type 2 diabetes by probing complementary biological pathways. The inflammation vulnerability marker, IVX, combines levels of GlycA, an integrated measure of *N*-acetyl methyl groups on acute-phase glycoproteins, with the concentration of small HDLPs, thereby providing a stable measure of chronic low-grade inflammation and impaired immune function [7]. Both of these NMR-derived biomarkers provide information that is not available from standard lipid panels or clinical examination. GlycA, by virtue of its composite nature, may be thought of as an integrated measure of inflammation over time, in contrast with the more volatile hsCRP level [20]; this is conceptually equivalent to the use of HbA_1c_ compared with random glucose. GlycA has been linked to incident cardiovascular events and mortality in populations without diabetes [36], acting as a summary of multiple glycoprotein elevations. Lower levels of small HDLPs may signify diminished vascular protection as they carry enzymes with antioxidant and anti-inflammatory functions [37]. We found that IVX was more strongly associated with mortality than hsCRP when included in the same model, although both contributed independently. This reflects the complexity of the impact that inflammation has on mortality and other health outcomes, which cannot be captured by a single biomarker.

MMX is calculated from circulating levels of citrate and BCAAs, with lower levels of the latter being linked previously to sarcopenia and altered protein metabolism [38, 39]. Sarcopenia develops more rapidly in people with type 2 diabetes [40], probably driven by insulin resistance, chronic inflammation, the presence of advanced glycation end-products, oxidative stress and mitochondrial dysfunction, and is associated with higher cardiovascular and mortality risk [41–43]. Complicating the understanding of how BCAA levels relate to sarcopenia and mortality risk in type 2 diabetes is the fact that obesity and insulin resistance are associated with higher BCAAs, but lower muscle mass and strength are linked to low BCAA levels [38, 44, 45]. Circulating BCAA levels exhibit stage-dependent associations in metabolic disease. In early insulin resistance and obesity, BCAAs are often elevated due to impaired catabolism. However, in more advanced stages, characterised by muscle loss and reduced anabolic reserve, lower BCAA levels may reflect sarcopenia and diminished metabolic resilience. The MMX framework is conceptually aligned with the latter phenotype, which may explain why low rather than high BCAA levels contribute to risk within this index.

In the FIELD trial participants, we found weak, non-significant associations of mortality with levels of all three BCAAs, contrasting with the greater contribution that low BCAA levels made to mortality risk in the CATHGEN cohort of coronary catheterisation patients that was used to develop the MVX [7]. The stronger BCAA and MMX associations in the CATHGEN cohort probably reflect a greater prevalence of metabolic malnutrition in that study, helping to explain its substantially higher short-term mortality incidence (17%) compared with the FIELD trial (7%). The results of our sensitivity analysis (ESM Table 2 and ESM Fig. 2), showing that MMX had a relatively stronger mortality association in higher-risk older participants (>70 years old), support this conjecture. Another contributing influence may be the relatively early stage of type 2 diabetes in the majority of FIELD participants, as evidenced by the short duration of known diabetes (geometric mean 4.3 years; SD factor 2.8) and 27% being treated through lifestyle measures alone in the overall cohort. The FIELD trial was a clinical trial with eligibility criteria that excluded individuals with major cardiovascular events within the last 3 months and those judged unsuitable for periodic clinical long-term follow-up. As such, individuals with frailty or overt malnutrition were probably under-represented. This restricted phenotypic variability may have attenuated associations between MMX and outcomes, contributing to the threshold pattern observed. Also, the higher prevalence of overweight and obesity in individuals with type 2 diabetes may modify the relationship between BCAAs and adverse outcomes. Direct comparisons of MVX/MMX with formal sarcopenia assessments (e.g. grip strength and imaging-derived muscle mass) in contemporary cohorts would help clarify the extent of overlap and incremental prognostic value.

By aggregating MMX and IVX, MVX encapsulates a ‘metabolic malnutrition–inflammation’ syndrome that simultaneously reflects muscle catabolism, immune activation and oxidative stress. The robust associations of MVX with both fatal and non-fatal cardiovascular outcomes, even after adjustment for hsCRP, glycaemia and traditional covariates, suggest that it captures risk pathways that are not fully addressed by routine clinical assays.

The incidence of sarcopenia in type 2 diabetes is 1.5–3 times higher than in individuals without type 2 diabetes, although the temporal relationship between muscle loss and diabetes onset remains debated [46]. Mendelian randomisation studies imply bidirectional causality between insulin resistance and sarcopenia [47], and emerging data suggest links between muscle wasting and diabetic nephropathy progression [48].

Importantly, the MVX components are amenable to lifestyle interventions. Both aerobic and resistance exercise have been shown to retard and even reverse sarcopenia in people with type 2 diabetes [49]. Exercise also decreased GlycA [50] and increased levels of small HDLPs [51], as did anti-inflammatory drug treatment in individuals with rheumatoid arthritis [50, 51]. Whether pharmacological therapies, such as fenofibrate, can similarly modulate these metabolomic signatures, and whether such modulation translates into improved clinical outcomes, remain topics for future research.

### Study strengths and limitations

Strengths of this study include use of the large and well-characterised FIELD cohort, which is broadly representative of the general population with type 2 diabetes, with many being early in their type 2 diabetes journey. The adjudicated nature of the clinical outcomes enhances the reliability of endpoint assessment, and provided an opportunity to consider both fatal and non-fatal cardiovascular and cancer outcomes. Additionally, NMR spectroscopy analyses demonstrated excellent reproducibility, with low coefficients of variation (<5%) across these measurements.

Limitations include the under-representation of certain high-risk groups, such as Indigenous populations and frailer participants, which may limit generalisability to these subgroups. While the in-trial follow-up period was modest, extended post-trial follow-up is underway, and will provide further outcome data over a longer time horizon. There was no reference group of participants without diabetes, but a wide range of degrees of diabetes (from those treated by diet alone to those treated with multiple oral medications plus insulin) and durations of diabetes (<1 to 36 years) were captured, and HbA_1c_ and duration of diabetes were found to be significant contributors to risk estimates. It is also important to note that many cardioprotective treatments that are now considered standard care, such as statins, SGLT2 inhibitors and GLP-1 receptor agonists, were not widely used during the FIELD trial period, and their use may alter these associations. Lastly, although NMR enables detailed metabolic profiling, its availability in routine clinical practice remains limited, potentially constraining clinical translation in the near future.

Sex was included as an adjustment variable in all multivariable models, and the MVX, IVX and MMX scores were calibrated to account for known sex-related differences in metabolite concentrations that are unrelated to mortality risk. However, we did not undertake sex-specific or sex-interaction analyses, as the primary objective of this report was to evaluate the prognostic performance of the predefined indices in the overall cohort. Consequently, although the present findings are applicable to both men and women included in the study, whether the magnitude of the associations differs by sex requires investigation in future reports of our studies.

### Conclusions

In this large, well-characterised cohort of individuals with type 2 diabetes, we demonstrated that novel metabolomic indices derived from NMR spectroscopy, particularly the MVX score, are strongly and independently associated with total mortality, cardiovascular mortality and non-fatal cardiovascular events. MVX showed the strongest and most consistent prognostic value, with a clear dose–response relationship across quintiles and in continuous analyses. Its predictive value persisted across a broad range of clinically relevant subgroups, surpassing traditional risk factors such as prior CVD history, and remained robust even after adjustment for hsCRP and other covariates. While IVX and MMX also showed associations with mortality, their contributions were more modest and less consistent, especially for non-fatal outcomes.

These findings suggest that composite metabolomic signatures such as MVX may provide incremental value for risk stratification among people with type 2 diabetes. Whether incorporation of such indices into clinical decision-making with treatment impacts improves patient outcomes remains to be determined. Future studies should evaluate the performance of these indices in additional contemporary cohorts, and explore whether risk-informed strategies guided by metabolomic profiling can meaningfully influence prevention pathways.

## Supplementary Information

Below is the link to the electronic supplementary material.

ESM (PDF 347 KB)

## Data Availability

De-identified participant data and relevant study variables supporting the findings of this study may be made available for academic use upon reasonable request, subject to the limitations of participant consent and approval by representatives of the FIELD trial steering committee. Requests should be directed to the corresponding author. Approved researchers will be required to enter into a data access agreement with the National Health and Medical Research Council Clinical Trials Centre, University of Sydney. Because of participant consent and governance restrictions, the full dataset cannot be deposited in a public repository.

## Abbreviations

BCAA: Branched-chain amino acid
GLP-1: Glucagon-like peptide-1
HDL-C: HDL-cholesterol
HDLP: HDL particle
hsCRP: High-sensitivity C-reactive protein
IVX: Inflammation vulnerability index
MMX: Metabolic malnutrition index
MVX: Metabolic vulnerability index
SGLT2: Sodium–glucose co-transporter-2
TC: Total cholesterol

## Authors’ relationships and activities

ACK reports receiving grants from the National Health and Medical Research Council, research support from Abbott, AbbVie, Amgen, Mylan, Aspen and Viatris, serving on an advisory committee for Viatris, receiving speaker honoraria from Novartis, Pfizer and Sanofi, and serving on a data safety monitoring board for Kowa. RJS reports receiving grants from the National Health and Medical Research Council, research support from Bayer, Bristol Myers Squibb, MSD, Amgen and Roche, and consulting fees from Fivephusion. DRS reports receiving grants from Regeneron, Amgen, Arrowhead, Ionis, Novartis and MSD, consulting fees from Amgen and Novartis, participation on advisory boards for the LIPID, FIELD and NOMAD studies, and serving as Clinical Committee Chair of the Australian Atherosclerosis Society. AJJ reports receiving grants from the National Health and Medical Research Council. JDO reports being a co-inventor on a patent (US11156621B2) owned by Labcorp relating to multivariable metabolic vulnerability index evaluations. The remaining authors declare that there are no other relationships or activities that might bias, or be perceived to bias, their work.
