# Supplementary material for: Indices of obesity, malnutrition and inflammation are strong predictors of mortality in adults with type 2 diabetes: the FIELD study
Source: Diabetologia. 2026 Sep 17;69(12):3533–47. doi: 10.1007/s00125-026-06848-6 (PMC13714137; doi:10.1007/s00125-026-06848-6)
Supplement: Supplementary file 1 [file 125_2026_6848_MOESM1_ESM.pdf]

Supplementary tables and figures

ESM Table 1.

Associations of MVX with fatal and non-fatal events among 5233 individuals at lower risk (with no prior macrovascular or microvascular disease at the FIELD study entry). MVX=metabolic vulnerability index. HRs and 95% CIs are from Cox proportional hazard regression models. The adjusted model includes the following covariates: fenofibrate treatment, age, sex, race (Caucasian vs other), smoking (current, ex, never), hypertension, BMI, total cholesterol, HDL-cholesterol, triglycerides, HbA1c, estimated glomerular filtration rate, hs-CRP and diabetes duration.

	**First Quintile**	**Second Quintile**	**Third Quintile**	**Fourth Quintile**	**Fifth Quintile**	**HR (95% CI) per SD**
**MVX score**	<=40.3	40.4 - 45.3	45.4 - 49.5	49.6 - 54.5	>=54.6	
**Total mortality**						
Events/*N* (%)	26/1257 (2.1%)	37/1161 (3.2%)	56/1096 (5.1%)	41/956 (4.3%)	64/763 (8.4%)	
HR (95% CI) - unadjusted	1·00 (reference)	1.53 (0.92-2.52)	2.44 (1.53-3.88)	2.01 (1.23-3.28)	3.98 (2.52-6.28)	1.61 (1.41-1.85)
HR (95% CI) – adjusted	1·00 (reference)	1.35 (0.81-2.24)	1.98 (1.24-3.18)	1.53 (0.92-2.53)	2.41 (1.46-3.97)	1.34 (1.16-1.56)
**CVD death**						
Events/*N* (%)	3/1257 (0.2%)	8/1161 (0.7%)	15/1096 (1.4%)	13/956 (1.4%)	20/763 (2.6%)	
HR (95% CI) - unadjusted	1·00 (reference)	2.84 (0.75-10.72)	5.64 (1.63-19.48)	5.49 (1.57-19.28)	10.72 (3.19-36.10)	1.96 (1.50-2.57)
HR (95% CI) – adjusted	1·00 (reference)	2.47 (0.65-9.37)	4.33 (1.24-15.15)	3.90 (1.08-14.05)	5.45 (1.50-19.77)	1.53 (1.13-2.07)
**Cancer death**						
Events/*N* (%)	16/1257 (1.3%)	23/1161 (2.0%)	34/1096 (3.1%)	23/956 (2.4%)	31/763 (4.1%)	
HR (95% CI) - unadjusted	1·00 (reference)	1.54 (0.82-2.92)	2.41 (1.33-4.37)	1.84 (0.97-3.48)	3.14 (1.72-5.75)	1.45 (1.21-1.74)
HR (95% CI) – adjusted	1·00 (reference)	1.34 (0.70-2.55)	1.92 (1.05-3.52)	1.42 (0.73-2.74)	2.01 (1.03-3.90)	1.25 (1.02-1.53)
**Non-fatal CVD**						
Events/*N* (%)	97/1257 (7.7%)	103/1161 (8.9%)	84/1096 (7.7%)	81/956 (8.5%)	84/763 (11.0%)	
HR (95% CI) – unadjusted	1·00 (reference)	1.14 (0.87-1.51)	0.98 (0.73-1.32)	1.06 (0.79-1.42)	1.40 (1.05-1.88)	1.08 (0.98-1.19)
HR (95% CI) – adjusted	1·00 (reference)	1.14 (0.86-1.51)	0.92 (0.68-1.25)	0.97 (0.71-1.33)	1.11 (0.80-1.55)	0.99 (0.89-1.10)
**Non-fatal cancer**						
Events/*N* (%)	57/1257 (4.5%)	47/1161 (4.0%)	43/1096 (3.9%)	48/956 (5.0%)	42/763 (5.5%)	
HR (95% CI) – unadjusted	1·00 (reference)	0.89 (0.60, 1.30)	0.86 (0.58, 1.28)	1.08 (0.74, 1.59)	1.21 (0.81, 1.80)	1.10 (0.96, 1.25)
HR (95% CI) – adjusted	1·00 (reference)	0.84 (0.57, 1.25)	0.84 (0.56, 1.25)	1.02 (0.68, 1.53)	1.09 (0.69, 1.71)	1.07 (0.92, 1.24)

ESM Table 2

Associations of MVX, IVX and MMX with total mortality in FIELD in ≤70 y.o. and >70 y.o. subgroups. IVX=inflammation vulnerability index. MMX=metabolic malnutrition index. MVX=metabolic vulnerability index. HRs and 95% CIs are from Cox proportional hazard regression models. The adjusted model includes the following covariates: fenofibrate treatment, age, sex, race (Caucasian vs other), smoking (current, ex, never), hypertension, BMI, total cholesterol, HDL-cholesterol, triglycerides, HbA1c, estimated glomerular filtration rate, prior CVD, hs-CRP and diabetes duration.

	**First Quintile**	**Second Quintile**	**Third Quintile**	**Fourth Quintile**	**Fifth Quintile**	**HR (95% CI) per SD**
**<=70 years old (*N*=8230)**						
**IVX score**	<=33.6	33.7 - 40	40.1 - 45.5	45.6 - 52.1	>=52.2	
Events/*N* (%)	45/1739 (2.6%)	67/1656 (4.0%)	84/1637 (5.1%)	117/1621 (7.2%)	171/1577 (10.8%)	
HR (95% CI) - unadjusted	1·00 (reference)	1.56 (1.07, 2.27)	1.96 (1.37, 2.82)	2.76 (1.96, 3.89)	4.18 (3.01, 5.81)	1.70 (1.56, 1.86)
HR (95% CI) - adjusted	1·00 (reference)	1.37 (0.94, 2.01)	1.52 (1.05, 2.20)	1.94 (1.36, 2.77)	2.45 (1.71, 3.51)	1.43 (1.28, 1.60)
**MMX score**	<=49.2	49.3 - 52.4	52.5 - 55.3	55.4 - 58.8	>=58.9	
Events/*N* (%)	87/1751 (5.0%)	103/1708 (6.0%)	77/1643 (4.7%)	90/1602 (5.6%)	127/1526 (8.3%)	
HR (95% CI) - unadjusted	1·00 (reference)	1.21 (0.91-1.61)	0.93 (0.68-1.26)	1.11 (0.83-1.49)	1.64 (1.25-2.16)	1.15 (1.06-1.26)
HR (95% CI) - adjusted	1·00 (reference)	1.10 (0.83-1.47)	0.87 (0.64-1.19)	0.94 (0.70-1.27)	1.37 (1.04-1.81)	1.09 (0.99-1.20)
**MVX score**	<=40.3	40.4 - 45.3	45.4 - 49.5	49.6 - 54.5	>=54.6	
Events/*N* (%)	45/1741 (2.6%)	60/1708 (3.5%)	96/1648 (5.8%)	98/1578 (6.2%)	185/1555 (11.9%)	
HR (95% CI) - unadjusted	1·00 (reference)	1.34 (0.91-1.98)	2.24 (1.57-3.19)	2.35 (1.65-3.34)	4.58 (3.30-6.34)	1.74 (1.59-1.90)
HR (95% CI) - adjusted	1·00 (reference)	1.14 (0.77-1.68)	1.74 (1.22-2.50)	1.63 (1.14-2.35)	2.55 (1.79-3.64)	1.42 (1.28-1.58)
**>70 years old (N=1320)**						
**IVX score**	<=33.6	33.7 - 40	40.1 - 45.5	45.6 - 52.1	>=52.2	
Events/*N* (%)	17/184 (9.2%)	22/245 (9.0%)	29/276 (10.5%)	47/287 (16.4%)	73/328 (22.3%)	
HR (95% CI) - unadjusted	1·00 (reference)	0.98 (0.52-1.85)	1.14 (0.63-2.08)	1.80 (1.03-3.13)	2.49 (1.47-4.22)	1.51 (1.30-1.76)
HR (95% CI) - adjusted	1·00 (reference)	0.86 (0.46-1.63)	1.03 (0.56-1.88)	1.44 (0.81-2.56)	1.63 (0.92-2.89)	1.28 (1.08-1.52)
**MMX score**	<=49.2	49.3 - 52.4	52.5 - 55.3	55.4 - 58.8	>=58.9	
Events/*N* (%)	15/165 (9.1%)	28/214 (13.1%)	41/260 (15.8%)	34/285 (11.9%)	70/396 (17.7%)	
HR (95% CI) - unadjusted	1·00 (reference)	1.44 (0.77-2.70)	1.77 (0.98-3.19)	1.32 (0.72-2.42)	1.94 (1.11-3.38)	1.14 (1.00-1.31)
HR (95% CI) - adjusted	1·00 (reference)	1.91 (0.96-3.80)	2.35 (1.22-4.51)	1.68 (0.86-3.27)	2.71 (1.45-5.08)	1.24 (1.07-1.43)
**MVX score**	<=40.3	40.4 - 45.3	45.4 - 49.5	49.6 - 54.5	>=54.6	
Events/*N* (%)	15/156 (9.6%)	17/223 (7.6%)	29/265 (10.9%)	43/312 (13.8%)	84/364 (23.1%)	
HR (95% CI) - unadjusted	1·00 (reference)	0.80 (0.40, 1.59)	1.10 (0.59, 2.05)	1.42 (0.79, 2.55)	2.44 (1.41, 4.23)	1.53 (1.32, 1.78)
HR (95% CI) - adjusted	1·00 (reference)	0.78 (0.39, 1.57)	1.04 (0.55, 1.95)	1.22 (0.67, 2.24)	1.86 (1.04, 3.33)	1.36 (1.15, 1.61)

ESM Figure 1

Variable contributions to overall model performance based on Chi-square statistic values from Cox proportional hazards models predicting total mortality. (a) The model includes clinical covariates with additional inclusion of IVX and MMX (C-statistic 0.737). (b) The model includes clinical covariates with inclusion of the metabolite components of IVX (GlycA, small HDLP) and MMX (isoleucine, valine, leucine, citrate) (C-statistic 0.738). Variables are ranked by their χ^2, highlighting the differential explanatory value of specific metabolic features. The number of participants included in multivariate models was 9425, after exclusion of 119 with missing hsCRP and 6 with missing BMI values.

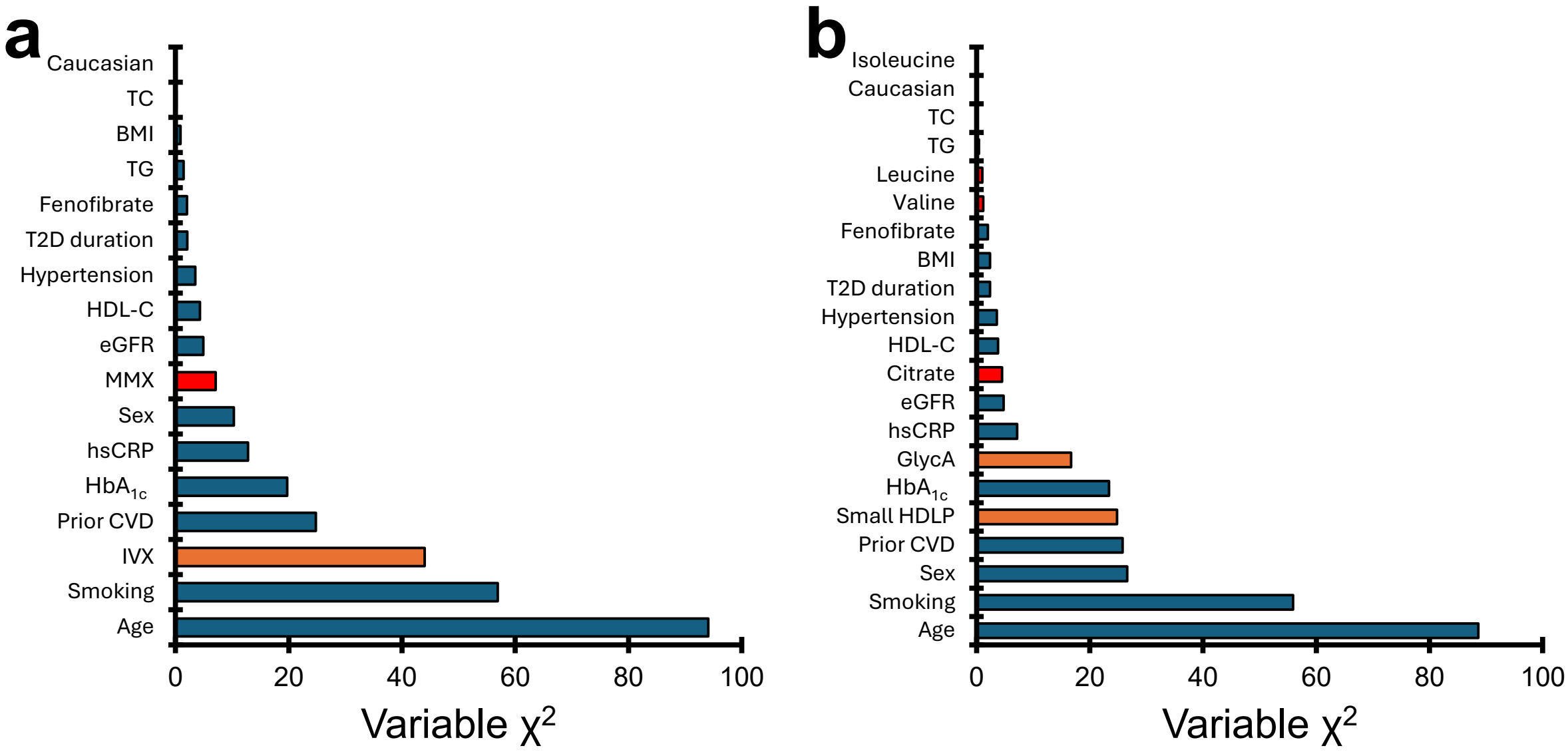

ESM Figure 2

Variable contributions to overall model performance from Cox proportional hazards models predicting total mortality in the younger ≤70 y.o. (a, c) and older >70 y.o. (b, d) participant subgroups. The younger subgroup (n=8122) had mortality incidence of 5.9% and mean age (range) of 61 years (49-70). The older subgroup (n=1303) had mortality incidence of 14.4% and mean age (range) of 73 years (71-75). (a, b) The models include clinical covariates with additional inclusion of IVX and MMX. (c, d) The models include clinical covariates with inclusion of the metabolite components of IVX (GlycA, small HDLP) and MMX (isoleucine, valine, leucine, citrate). Variables are ranked by their χ^2, highlighting the differential explanatory value of specific metabolic features. MMX and its metabolite components are shown to contribute substantially more to mortality prediction in the older participant subgroup.

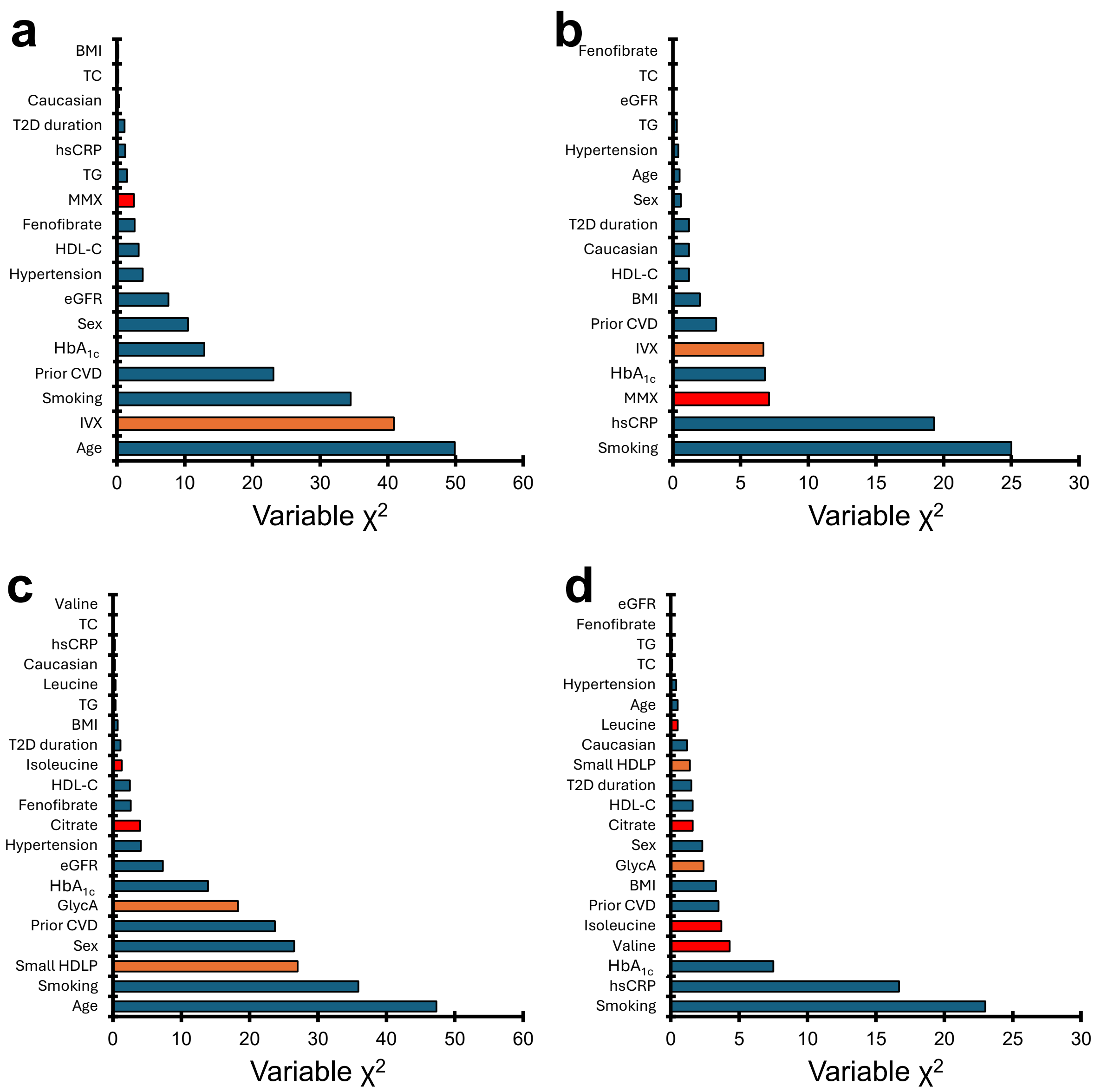

ESM Figure 3.
Heatmap of frequency distributions across IVX and MMX quintiles. Each cell represents the number of participants classified into the corresponding quintile of IVX (rows) and MMX (columns). While the Pearson correlation coefficient was low ($r = 0.03$, $p = 0.009$), indicating minimal linear relationship between the two indices, the Chi-square test revealed a statistically significant non-random association between their joint distributions ($p = 0.0081$). This suggests that the overlap between IVX and MMX quintiles differs from what would be expected under independence.

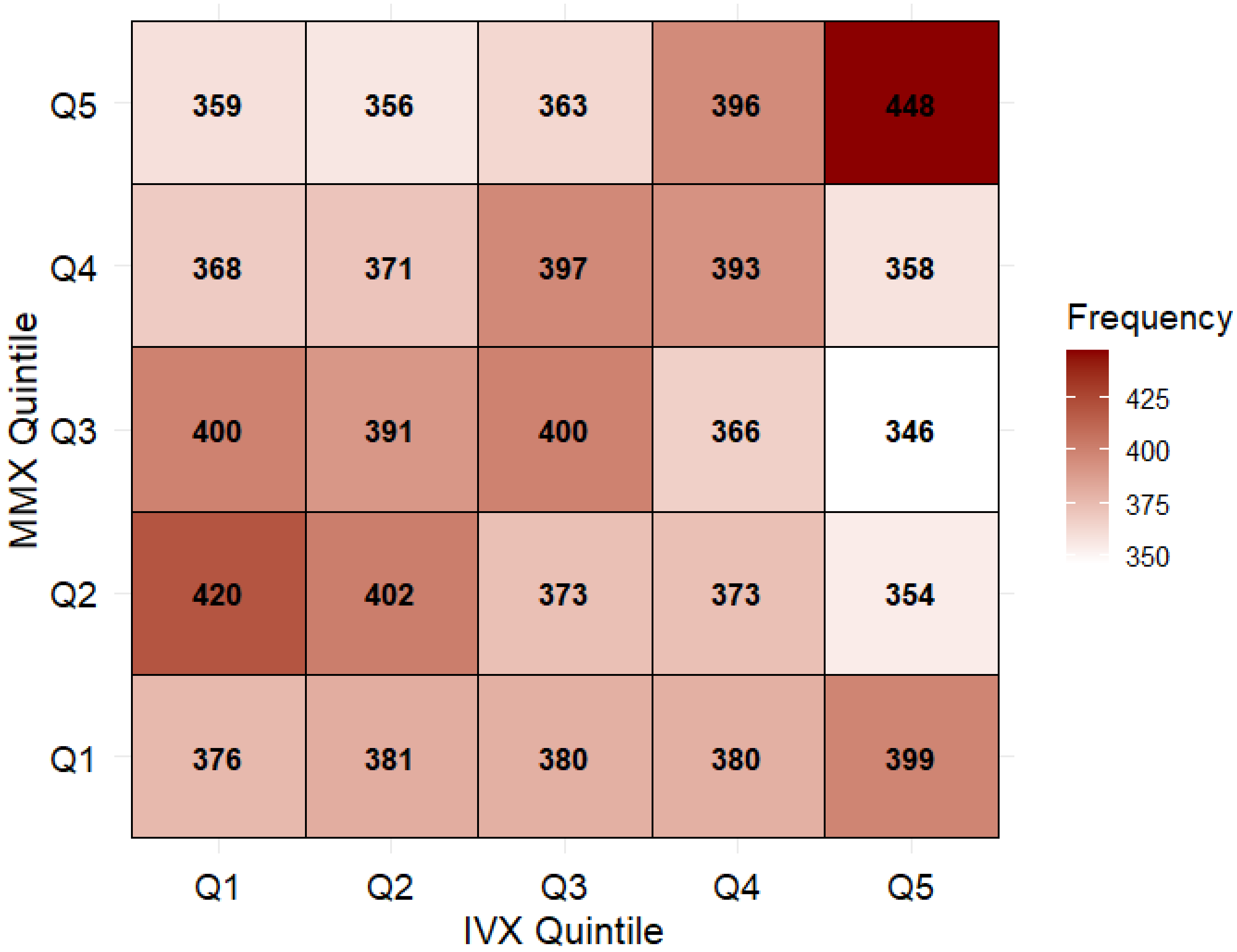

FIELD Study Group members

Management committee

Executive committee members: P Barter, J Best, A Keech (study chairman and principal investigator), R Scott, R J Simes, M-R Taskinen
Other: P Colman, M d'Emden, T Davis, P Drury, C Ehnholm, P Glasziou, D Hunt, YA Kesäniemi, M Laakso, D Sullivan, M Whiting, J-C Ansquer, B Fraitag (non-voting sponsor representatives).

Outcomes assessment committee

N Anderson, G Hankey, D Hunt (chairman), S Lehto, S Mann, M Romo, L P Li (outcomes officer, in attendance).

Safety and data monitoring committee

C Hennekens, S MacMahon (chairman), S Pocock, A Tonkin, L Wilhelmsen; P Forder (unblinded statistician, in attendance).

Site principal investigators

Australia: H Akauola, F Alford, P Barter, I Beinart, J Best, S Bohra, S Boyages, P Colman, H Connor, D Darnell, T Davis, P Davoren, F Lepre, F De Looze, M d'Emden, A Duffield, R Fassett, J Flack, G Fulcher, S Hamwood, D Harmelin, R Jackson, W Jeffries, M Kamp, L Kritharides, L Mahar, V McCann, D McIntyre, R Moses, H Newnham, G Nicholson, R O'Brien, K Park, N Petrovsky, P Phillips, G Pinn, D Simmons, K Stanton, B Stuckey, D R Sullivan, M Suranyi, M Suthers, Y Tan, M Templer, D Topliss, J H Waites, G Watts, T Welborn, D Wilson, R Wyndham.
Finland: H Haapamäki, A Kesäniemi, M Laakso, J Lahtela, H Levänen, J Saltevo, H Södervik, M-R Taskinen, M Vanhala
New Zealand: J Baker, A Burton, P Dixon, J Doran, P Drury, P Dunn, N Graham, A Hamer, J Hedley, P Manning, I McPherson, S Morris, C Renner, R Scott, R Smith, M Wackrow, S Young.

Co-investigators and site coordinators

Australia: J Alcoe, C Allan, J Amerena, C Anderson-Stanford, N Arnold, T Arsov, D Ashby, C Atkinson, L Badhni, M Balme, D Barton, B Batrouney, C Beare, T Beattie, J Beggs, C Bendall, C Bendall, A Benz, D Black, A Bond, R Bradfield, J Bradshaw, S Brearley, D Bruce, J Burgess, M Callary, J Campbell, H Carr, K Chambers, J Chow, S Chow, K Ciszek, P Clifton, P Clifton-Bligh, V Clowes, P Coates, C Cocks, S Cole, D Colquhoun, M Correcha, S Coverdale, M Croft, J Crowe, W Davis, J Dunn, S Edwards, R Elder, S El-Kaissi, L Emery, M England, O Farouque, M Fernandez, B Fitzpatrick, N Francis, P Francis, P Freeman, A Fuller, V Gaylard, C George, C Gillzan, C Glatthaar, J Goddard, V Grange, T Greenaway, J Griffin, A Grogan, S Guha, J Gustafson, P S Hamblin, I Hamilton-Craig, T Hannay, C Hardie, A Harper, G Hartl, A Harvey, S Havlin, K Haworth, P Hay, L Hay, B Heenan, R Hesketh, A Heyworth, M Hines, G Hockings, A Hodge, L Hoffman, L Hoskin, M Howells, D Hunt, A Hunt, W Inder, D Irvine, D Jackson, C Jang, K Kearins, P Kee, J Keen, D Kilpatrick, J Kindellan, M Kotowicz, E Lahz, A Lassig, M Layton, S Lean, H Legg, E Lim, F Long, L Lucas, D Ludeman, C Ludeman-Robertson, M Lyall, L Lynch, C Maddison, B Malkus, A Marangou, F Margrie, N Martyn, K Matthiesson, J Matthiesson, S Maxwell, V Maxwell, K McCarthy, A McElduff, H McKee, J McKenzie, K McLachan, P McNair, M Meischke, A Merkel, C Miller, E Morrison, A Morton, W Mossman, A Mowat, J Muecke, P Murie, S Murray, P Nadorp, S Nair, J Nairn, A Nankervis, K Narayan, N Nattrass, J Ngui, S Nicholls, V Nicholls, C Nolan, J A Nye, E Nye, D O'Neil, M O'Neill, S O'Rourke, J Pearse, C Pearson, L Pittis, D Playford, L Porter, L Porter, R Portley, M Powell, C Preston, S Pringle, J Raffaele, G Ramnath, J Ramsden, G Rayment, S Roffe, S Rosen, G Ross, Z Ross, J Rowe, D Rumble, S Ryan, N Sachithanandan, J Sansom, L Sefert, E Shanahan, J Shepherd, G Sherman, R Siddall, D Silva, S Simmons, R Simpson, A Sinha, R Slobodniuk, M Smith, P Smith, S Smith, V Smith-Orr, L Socha, T Stack, K Steed, K Steele, J Stephensen, P Stevens, G Stewart, C Strakosch,

M Sullivan, S Sunder, J Sunderland, E Tapp, J Taylor, D Thorn, A Tolley, D Torpy, G Truran, F Turner, J Turner, S Varley, J Wallace, J Walsh, J Walshe, G Ward, B Watson, J Watson, A Webb, F Werner, E White, A Whitehouse, N Whitehouse, S Wigg, E Wilmshurst, G Wittert, B Wong, M Wong, S Worboys, S Wright, S Wu, J Yarker, M Yeo, K Young, J Youssef, R Yuen, H Zeimer, R W Ziffer.
Finland: A Aura, A Friman, J Hanninen, J Henell, A Hiukka, N Hyvärinen, M Ikonen, A Itkonen, A Jantunen, A Jarva, T Jerkkola, V Jokinen, J Juutilainen, H Kähkönen, T Kangas, P Karja-Koskenkari, L Karjalainen, M Karttunen, P Kauranen, S Kortelainen, H Koukkunen, L Kumpulainen, T Laitinen, M Laitinen, S Lehto, R Lehto, E Leinonen, M Lindströn-Karjalainen, A Lumiaho, J Mäkela, K Makinen, L Mannermaa, T Mård, M Metso, J Miettinen, V Naatti, S Paavola, P Pajunen, S Pöykkö, A Pulkkinen, J Ripatti, S Ruotsalainen, A Salo, M Siiskonen, A Soppela, J Starck, I Suonranta, A Toivonen, L Ukkola, K Valli, S Vehkavaara, J Virolainen.
New Zealand: P Allan, W Arnold, W Bagg, K Balfour, P Ball, B Ballantine, C Barker, C Barker, F Bartley, E Berry, G Braatvedt, A Campbell, T Clarke, R Clarke, A Claydon, S Clayton, P Cresswell, R Cutfield, J Delahunt, A Dissnayake, C Eagleton, C Ferguson, C Florkowski, D Fry, P Giles, M Gluyas, C Grant, P Guile, M Guolo, P Hale, M Hammond, M Hammond, P Healy, M Hills, J Hinge, J Holland, B Hyne, A Ireland, A Johnstone, S Jones, G Kerr, K Kerr, M Khant, J Krebs, L Law, B Lydon, K MacAuley, R McEwan, P McGregor, B McLaren, L McLeod, J Medforth, R Miskimmin, J Moffat, M Pickup, E Reda, C Ross, A Ryalls, D Schmid, N Shergill, H Snell, L Stevens, A Waterman, V Watts.

Coordinating centre teams

NHMRC clinical trials centre, Sydney: S Blakesmith, K Jayne, E Keirnan, P Newman, G Ritchie, A Rosenfeld (project directors), E Beller, P Forder, V Gebski, A Pillai (study statisticians), S Copt, S Heritier, A Kirby, R O'Connell (validation statisticians), C Sutton (data systems manager), C Anderson, T Biddle, S-Y Chan, S Czyniewski, A Dobbie, S Doshi, A Dupuy, S Eckermann, M Edwards, N Fields, K Flood, S Ford, C French, S Gillies, C Greig, M Groshens, J Gu, M Guo, Y Guo, W Hague, S Healey, L Hones, Z Hossain, M Howlett, J Lee, L-P Li, F Liang, T Matthews, J Micallef, I Minns, S Mulray, A Nguyen, F Papuni, A Patel, J Pearse, R Pike, M Pena, K Pinto, D Schipp, J Schroeder, B Sim, L Snaith, T Sourjina, R Taylor, P Vlagsma, S Walder, R Walker, J Zhang, B Zhong, A Keech (deputy director), R J Simes (director).
Helsinki project office: A Kokkonen, A Leppämäki, P Narva, A Salo, A-M Syrjänen, M-R Taskinen (director).
Christchurch project office: C Lintott, R Scott (director).

Central laboratories

SouthPath Laboratories, Flinders Medical Centre, Adelaide, Australia: L Clark, J Leach, M Pejakovic, R Tirimacco, M Whiting. National Public Health Institute Laboratories,
Helsinki, Finland: C Ehnholm, M Ikonen, M Kajosaari, L Råman, J Sundvall, M Tukianen.

Laboratoires Fournier SA liaison

Dijon: J C Ansquer, B Fraitag, D Crimet, F Aubin, F Golly, I Sirugue, C Foucher, K Le Malicot.
Sydney: P Aubonnet.
